# Virus-Incorporated Biomimetic Nanocomposites for Tissue Regeneration

**DOI:** 10.3390/nano9071014

**Published:** 2019-07-15

**Authors:** Iruthayapandi Selestin Raja, Chuntae Kim, Su-Jin Song, Yong Cheol Shin, Moon Sung Kang, Suong-Hyu Hyon, Jin-Woo Oh, Dong-Wook Han

**Affiliations:** 1 Monocrystalline Bank Research Institute, Pusan National University, Busan 46241, Korea; 2 Department of Nanofusion Technology, College of Nanoscience & Nanotechnology, Pusan National University, Busan 46241, Korea; 3 Department of Cogno-Mechatronics Engineering, College of Nanoscience & Nanotechnology, Pusan National University, Busan 46241, Korea; 4 Department of Medical Engineering, Yonsei University, College of Medicine, Seoul 03722, Korea; 5 Joint Faculty of Veterinary Medicine, Kagoshima University, Kagoshima 890-8580, Japan

**Keywords:** virus, tissue regeneration, biomimetic nanocomposites, phage display

## Abstract

Owing to the astonishing properties of non-harmful viruses, tissue regeneration using virus-based biomimetic materials has been an emerging trend recently. The selective peptide expression and enrichment of the desired peptide on the surface, monodispersion, self-assembly, and ease of genetic and chemical modification properties have allowed viruses to take a long stride in biomedical applications. Researchers have published many reviews so far describing unusual properties of virus-based nanoparticles, phage display, modification, and possible biomedical applications, including biosensors, bioimaging, tissue regeneration, and drug delivery, however the integration of the virus into different biomaterials for the application of tissue regeneration is not yet discussed in detail. This review will focus on various morphologies of virus-incorporated biomimetic nanocomposites in tissue regeneration and highlight the progress, challenges, and future directions in this area.

## 1. Introduction

### 1.1. Emerging Trends in Tissue Regeneration

Tissue engineering is a part of the regenerative medicine field, which emphasizes the fabrication of various functional biological constructs to reduce the increased demand for donor organs [1]. The shortage of organ donors and the increased number of people undergoing transplantation have necessitated the development of effective biomimetic materials adopting advanced technologies [2]. The aim of the field is to harness nature’s ability to treat the damaged tissues., ensuring biocompatibility and supporting cellular biological events. When muscles are damaged by incidents such as illness, accidents, and microbial invasion, they lose integrity for healthy functioning at the cellular level and subsequently follow a cascade of biochemical events, including hemostasis, inflammation, proliferation, and maturation, to restore integrity [3]. However, the first ever immediate response after an injury is the production of reactive oxygen species by NADPH oxidase before inflammatory reaction [4]. Moreover, the time consumption to retain normal function in the dysfunctional organ is dependent on one’s age and the seriousness of the damage. When tissue fails in the ability for self-regeneration, especially in a pathological condition, the external application of a scaffold becomes inevitable [5].

Stainless steel was first implanted as an artificial hip in 1929, which paved the way for the researchers to design biomimetic materials to replace body organs [6]. However, tissue and organ failure were considered as major medical complications until 1954, as there were not enough remedies to treat dysfunctional organs. The Nobel Laureate in medicine, Joseph Murray, transplanted a healthy kidney to a genetically identical twin brother successfully [1]. Following this historical incident, the researchers began to utilize cell structures to treat damaged tissues. These structures are autografts, isografts, allografts, and xenografts, which are biological tissues harvested from a patient’s organ, a genetically identical twin, a genetically non-identical individual of the same species, and a donor of a different species, respectively [7]. The autograft has some advantages over other forms of grafts, with no rejection concerns. However, it requires a secondary procedure for the reconstructive surgery and faces donor site complication.

Meanwhile, isografts, allografts, and xenografts express slight or significant degrees of rejection as the Major Histocompatibility Complex (MHC) recognizes them as foreign antigens causing cytotoxic T-cell-mediated demolition. Unlike autografts, other forms of grafts eliminate the need for the secondary procedure without the donor site complications [8]. Though researchers are involved in finding therapeutic techniques, such as immunosuppressive medications, infectious prophylaxis, and DNA-based tissue typing, graft-versus-host disease (GVHD) remains a hurdle limiting the use of grafts in tissue regeneration [9].

The extracellular matrix (ECM) is an important component in tissues, which has the intrinsic cues to provide sites with all their cellular activities, including migration, orientation, shape, plasticity, and cell–matrix and cell–cell interactions [10]. The ECM is a complex three-dimensional network comprising proteins, such as collagen, elastin, and laminin, proteoglycans, and glycoproteins [11]. It acts as a glue and platform to bind cells together with the connective tissues. The decellularized extracellular matrix (DECM), a water-insoluble matrix, can be prepared from any organ or tissue by removing cellular components from ECM. In recent years, DECM has been used as a scaffold to hold the cells together and maintain integrity during wound healing processes [12]. Over the past two decades, the researchers have been inclined towards the preparation of artificial biomimetic grafts from the naturally available biomacromolecules, synthetic polymers, and ceramic materials, to replace tissue grafts and DECM. The remarkable features of artificial grafts are that they are economically inexpensive, their ease of preparation and storage, biocompatibility, and the tuning of the physicochemical and biological properties [13]. The most widely studied natural polymers include collagen, chitosan, hyaluronic acid, carboxymethyl cellulose, alginate, and silk fibroin, which have been reported to trigger a less immunogenic reaction in the human body, while the synthetic polymers, such as polycaprolactone, polyvinyl alcohol, nylon, and polyphosphazene, provide longer shelf life. To obtain combinatorial properties, nowadays researchers utilize a blend of polymers and their derivatives [14,15]. Ceramic and bioglass materials exhibit osteoconductive properties, showing remarkable mechanical strength and resistance to deformation, and hence, they have been widely used for bone tissue regeneration [16]. While designing a synthetic biomimetic scaffold, it is a requisite to create a benign microenvironment in a way that mimics the ECM niche for the application of effective tissue regeneration.

In the past ten years, researchers have explored a variety of biomimetic nanocomposites by incorporating bioactive molecules, such as growth factors, cytokines, genes, antibiotics, and anti-inflammatory drugs within the scaffold to increase tissue regeneration potential [17,18]. As the direct administration of therapeutic medications is not effective, leading to overdose toxicity and short time exposure to biological tissue, these biomimetic nanocomposites were much appreciated by chemists, biologists, and nanotechnologists [19]. Mostly, the bioactive molecules are cross-linked with the primary polymeric component by physical, chemical, and enzymatical treatment with or without the aid of cross-linkers and their release from the matrix is adjusted for either immediate, sustained, or slow release, according to the desired site of the target tissue. The drug release rate from the biomaterial majorly involves simple diffusion, erosion, or degradation of the matrix and swelling of the polymeric scaffold [5]. After the advent of nanotechnology, the nanoparticles were entrapped into the scaffold to exploit their medicinal properties. A composite of collagen and silver nanoparticles has been used to augment the burn tissue repair process [20]. Mieszawska et al. studied a composite film composed of silk and nanoclay to serve as a supportive biomaterial to improve bone tissue regeneration [21]. The in vivo effects of reduced graphene oxide and hydroxyapatite nanocomposite powders were investigated on critical-sized calvarial defects in a rabbit model and it was reported that the nanocomposite stimulated osteogenesis and enhanced bone formation without inflammatory responses [22]. Our research group also studied the influence of graphene oxide dispersed into a polylactic-co-glycolic acid (PLGA) electrospun nanofiber towards stimulation of myogenesis and enhanced vascular tissue regeneration [23,24]. The nanoparticles with diameters in the range of 50–700 nm acted as therapeutic drug carriers to pass through the capillaries into cells, facilitating the regeneration of new tissues [5,25].

**Figure 1:**
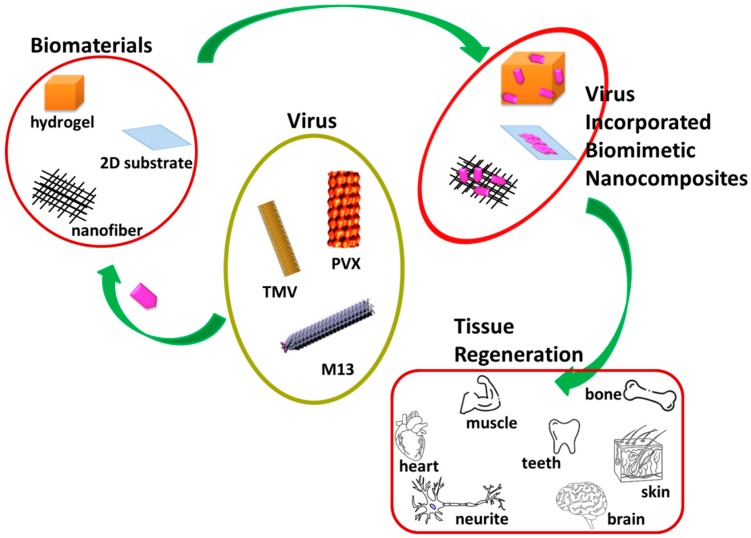
A compendium of tissue regeneration functionality of various virus incorporated biomimetic nanocomposites. Plant-based viruses Tobacco Mosaic Virus (TMV) and Potato Virus X (PVX) and bacteriophage M13 were shown. Reproduced with permission from [31]. Copyright Elsevier, 2010.

Cell-laden scaffolds have also received attention among researchers aiming to achieve a tissue-imitating engineered graft. Kizilel et al. encapsulated pancreatic islets into nano-thin polyethylene glycol coating for enhanced insulin secretion [26]. Yoon et al. fabricated a three-dimensional layered structure using the blend of collagen epidermal keratinocytes and dermal fibroblasts to progress migration and proliferation of keratinocytes and fibroblasts during the skin repair process [27]. Within this context, microbe-based biomimetic materials have appeared as an emerging trend in tissue regeneration in recent times. Virus-based biomaterials have many biomedical applications, including cancer markers, antibacterial materials, drug carriers, and tissue regeneration [28]. Not only do they encapsulate and release the therapeutic agents to the target site, but the morphology of biomaterials also plays a pivotal role in altering physicochemical and biological properties. In this review, we have focused on summarizing the impacts of various virus-incorporated biomimetic nanocomposites with different morphologies, such as nanoparticles, nanofibers, hydrogels, and organic-inorganic hybrids, in the field of tissue regeneration. The same has been schematically shown in Figure 1. The nanoparticles that have a large surface area to volume have proven their effectiveness with long-term functionality and stability in the biological milieu. The nanoparticles can diffuse across the cell membrane and interact with cellular biomacromolecules residing inside the cell [20]. Remarkably, the hydrogel provides wettability and cell migration, while the nanofibrous matrix ensures air permeability and mechanical properties in tissue regeneration [29,30].

### 1.2. Remarkable Properties of Medicinally Valuable Viruses

Not all viruses cause infectious diseases in the human body. Viruses can be classified as lytic, temperate, or lysogenic based on the level of adverse effects produced in its host [32]. During infection, lytic phages kill the host bacteria, triggering the release of progeny. Lysogenic phages do not affect the host cell and infection occurs with replication of the phage genome but not the host bacterial genome. Temperate phages reside in host bacteria for amplification with no lysis, however some phages, including the λ phage, exceptionally, have both categories and thrive following either lytic or lysogenic cycles. The lytic phages, including T1–T7, contain a head and flexible tail but lack filaments. The T7 phage belongs to the Podoviridae family and structurally has an icosahedral head and a short tail. They were reported to lyse the host cell within a minute by secreting the lysozyme enzyme [33]. Professionally, T4 phages have found applications in food preservation, antibiotics, detection of bacteria, DNA and protein packing systems, and DNA-based vaccines. The literature reports revealed that Podovirus P22 assisted the assembly of cadmium sulfide nanocrystals to improve photosensitization in tissue imaging [34]. The filamentous phages, including Ff, f1, M13, N1, and Ike, are the examples of temperate phages. As they can act as a template for the synthesis of nanomaterials, the general applications of temperate phages are huge compared to lytic phages [33].

Virus-based biomimetic materials are generally derived from plant viruses and bacteriophages, as they rarely generate harmful side effects in human beings. Generally, bacteriophages (excluding fd and M13) are categorized into filamentous type of viruses, follow a non-lytic mode to infect and thrive in bacteria. It has been reported that these phages do not consist of mammalian promoter sequences in their genome, and hence, do not instigate dreadful human diseases [35]. In the human body, bacteriophages are present abundantly in the gut, bladder, and oral cavity, functioning to shape bacterial metabolisms and populations of microbial communities. It has been described that the potential role of phages increases from childhood to adulthood [36]. The monodispersed phages can self-assemble themselves into hierarchically ordered structures, such as rope-like bundles and liquid crystals. The protein surface can be modified either by covalent and non-covalent interactions or genetic alterations [35]. These unique properties have prompted the researchers to take a long stride in utilizing the phage-based biomaterials towards a wide range of biomedical applications, including biomedical imaging, drug delivery, biosensors, tissue regeneration, energy, and catalysis [37].

Owing to economically inexpensive, large scale production, ease of manipulation, and stability against a wide range of pH and temperature, a variety of phage-based biomimetic nanocomposites have been constructed for the application of effective tissue regeneration [38]. As far as morphology is concerned, a typical phage has a diameter of 68 A° and a length in the range of 800–2000 nm. The circular single-stranded DNA (ssDNA) of the phage encodes 10 genes containing 5000–8000 nucleotides, which encode a highly ordered major coat protein (p8) located around the center of phage, two minor coat proteins (p7 and p9) at one end, and two others (p3 and p6) at another terminal portion of the phage. The helical arrays of major coat proteins assemble to form the capsid shell. Generally, minor coat proteins display larger sized peptides than the major coat protein (p8) [39]. The major coat protein, p8, of M13 phage, has different segments, such as the N-terminal amphipathic, hydrophobic transmembrane (TM), and DNA binding segments. The small residues (Gly, Ala, and Ser) present on these segments have been reported to be involved in helix–helix axial and lateral interactions, which facilitate extrusion of the virion from the membrane during assembly, and hence have been known as conserved regions in the DNA sequence. Fiber diffraction and spectroscopic data show that M13 differs from fd at the 12th residue, where M13 replaces Asp of fd with Asn [40]. Filamentous phages are defined as non-enveloped bacterial viruses, having some properties in common, namely, life cycles, organization, and morphology.

The ssDNA has a left-handed helix structure possessing strong interactions with the positively charged inner surface of the capsid shell. The diffraction pattern studies classified filamentous phages into two distinct groups. Class I symmetry group consists of fd, M13, If1, and IKe, which are consistent with 5-fold symmetry. Class II symmetry group includes Pf3, Pf1, and Xf, wherein the helices are arranged with a rising per monomer of about 3.0 A° [39]. The aligned solid-state NMR studies proved that fd has O-P-O phosphate linkages in an ordered manner, whereas Pf1 did not possess such linkages [41]. According to NMR studies, phage fd has strong electrostatic interactions between the negatively charged phosphate backbone of the ssDNA nucleotide and two of four positively charged amino acid residues present at the C-terminal portion of the major coat protein, which is attributed to stabilization of the DNA core structure. The literature reports revealed that M13 and IKe showed similarity in π–π interactions between the residues of Tyr9 of one p8 and Tyr29 of an adjacent p8 [42].

Infection of E. coli by phage is initiated by the attachment of N-terminal amino acids of p3, which is present in the specialized threadlike appendage, F pilus. Subsequently, the coat protein of the phage dissolves onto the envelope of the host, which allows the only ssDNA into the cytoplasm. The host machinery synthesizes a complementary DNA strand with the involvement of two virally encoded proteins, p2 and p10, which leads to the formation of a double-stranded replicative form. The replicative form acts as a template to transcript phage genes for the synthesis of progeny ssDNAs. These progeny phage particles discharge from the bacterial cell envelope through the membrane pore complex, acquire coat proteins from the membrane, and appear as mature virions. The fact is that the infected cells undergo division at a slower rate than the uninfected cells [39].

In recent times, the researchers have sought to explore multifunctional phage-based biomaterials by precisely adjusting the surface chemistry of phage nanofibers. Covalent, non-covalent, and genetic modifications of phage coat proteins have been described comprehensively by the researchers. The genetic modification of phage coat proteins would display various foreign peptides with different functional groups at the side wall and the two termini of the phage. The endogenous amino acids of phage coat proteins are genetically combined with the foreign amino acid sequence in order to form a hybrid fusion protein, which is incorporated into phage particles and released from the cell subsequently. As a result, the foreign peptide is displayed on the surface of the phage coat protein [35]. The phage display is generally specified after N-terminal modification in its respective coat proteins. For example, if the N-terminus of p3 of the phage undergoes modification, the resulting phage is designated as a p3 display. When two or more coat proteins are controlled for modification in the same phage, then they can be known as double display, and so on [43]. In the phage coat protein, the carboxylates of aspartic and glutamic acid residues, the amine of lysine, and the phenol of tyrosine are the majorly available functional entities for the chemical modification. Introducing aldehyde into the reactive amine group has been involved in a wide range of bio-conjugation reactions, whereas the cross-linkage of p-azidophenylalanine has provided an azide handle on the phage surface, which can be easily modified for further reactions [44,45]. The EDC treatment has been helpful in cross-linking the reactive carboxylate groups with amine-functionalized moieties in phage proteins [46]. Strong nucleophile selenocysteine has been successfully genetically incorporated into phage protein using an opal stop, codon suppressing mRNA [47].

The phage-display library, with a heterogeneous mixture of phages carrying different foreign DNA insert, was created for selective binding of phage proteins with the target ligands, such as polymers, proteins, organic and inorganic crystals, small molecules, such as trinitrotoluene, and cells [48,49,50,51]. Among the phage-display libraries, the reports of p3 and p6 libraries have been well documented in research publications. Conventionally, research studies have adopted the biopanning method to find extensive use of phage particles in tissue regeneration. Biopanning is a typical technique to form a population of enriched phage-displayed peptides and specifically identify a target binding peptide [52]. According to this selection procedure, initially, a phage-display random library is incubated with the targets. Subsequently, the non-bound phage particles are eliminated with the help of detergent solubilized buffer. The target-bound phage particles are then eluted using a specialized buffer maintaining acidic pH around 2.2, and the amplification process is followed by infection of host bacteria. The resulting amplified phages form a newly enriched sub-library with more specificity to interact with the targets. The procedure is repeated several times until a only few desired peptides are predominantly available in the sub-library [53]. In the subsequent section, we will investigate the contribution of plant virus and phage-based biomimetic nanocomposites in the field of tissue regeneration.

## 2. Different Morphologies of Virus-Incorporated Biomimetic Nanocomposites in Tissue Regeneration

### 2.1. Virus-Based Nanoparticles

Many plants and phage-based viral nanoparticles have been employed so far for tissue regeneration. Plant viral nanoparticles are mono-dispersed, meta-stable, and structurally uniformed [54]. Li revealed that when the virus-based nanoparticle is more robust, the functional nanostructure is more stable, but at the same time they might be harmful to the encapsulated cargo [55].

**Figure 2:**
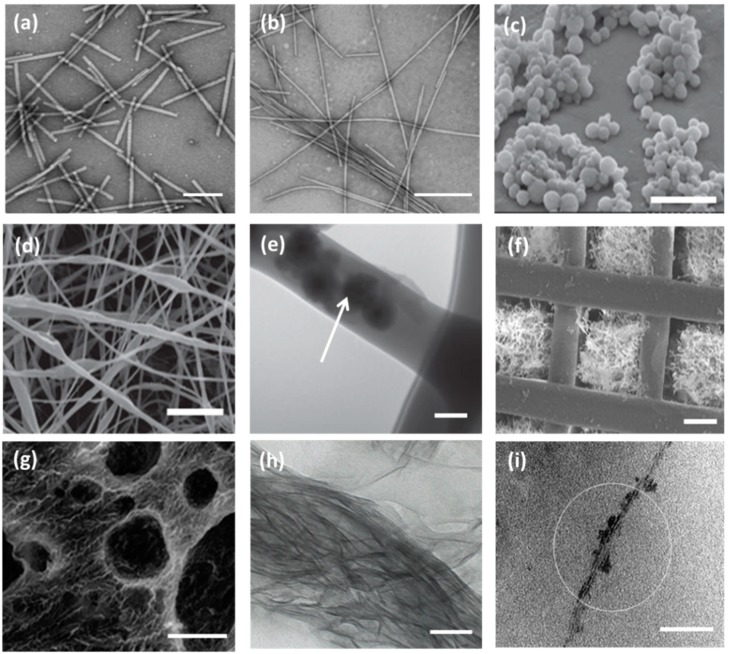
TEM micrographs of wild type Tobacco Mosaic Virus (TMV) nanoparticles, scale bar 200 nm (**a**) and TMV/PANI/PSS nanofiber (**b**) generated by flow assembly method, scale bar 500 nm. Reproduced with permission from [56]. Copyright American Chemical Society, 2015. (**c**) SEM micrograph of freeze-dried capsules of T4 bacteriophage/alginate water in oil emulsion in chloroform, scale bar 5 μm. (**d**) SEM and TEM micrographs (**e**) of PEO electrospun nanofiber containing T4 bacteriophage/alginate have been shown scale bars of 10 μm and 100 nm, respectively. The arrow indicates the presence of T4/alginate into the nanofiber. Reproduced with permission from [57]. Copyright John Wiley and Sons, 2013. (**f**) SEM image of the 3D printed bioceramic bone scaffold consisting of biphasic calcium phosphate with pores filled with a matrix of chitosan and RGD phage, scale bar 200 μm. Reproduced with permission from [58]. Copyright John Wiley and Sons, 2014. (**g**) SEM micrograph of porous alginate hydrogel containing TMV particles displaying interconnected channels and macropores, scale bar 100 μm. Reproduced with permission from [59]. Copyright American Chemical Society, 2012. (**h**) The TEM image of a mineralized E8-displaying phage bundle formed after 90 h incubation in the solution containing calcium and phosphate ions, scale bar 100 nm. Reproduced with permission from [60]. Copyright John Wiley and Sons, 2010. (**i**) The TEM image of the HAP-fd phage bundle with scale bar 100 nm. The circle indicates the presence of hydroxyapatite nanoparticles in the fibrous structure. Reproduced with permission from [61]. Copyright American Chemical Society, 2010.

Though the unmodified TMV nanoparticles have the potential to accelerate osteogenic differentiation in adult stem cells, the lack of affinity to the mammalian cell surface diminishes the cell adhesion property. Hence, the researchers opt for either genetic or chemical modification in viral nanoparticles in order to increase the cell binding capacity and find versatile biomedical applications. Sitasuwan et al. [62] modified the surface of a TMV nanoparticle by coupling azide-derivatized Arg-Gly-Asp-(RGD) tripeptide with tyrosine residues through Cu (I) catalyzed azide-alkyne cycloaddition reaction. When incorporated into the artificial scaffold, the RGD peptides overexpressed on ECM increase initial cell attachment by binding integrin receptors. The spacing between RGD motifs alter biological events, such as fibroblast adhesion and spreading (˂440 nm), focal adhesion assembly (˂140 nm), and induction of stress fiber formation (˂60 nm). Owing to lack of mammalian cell infectivity, cost-effectiveness, and highly uniform size, plant viral nanoparticles have gained attention among nano and biomedical researchers. The Tobacco Mosaic Virus (TMV) constitutes a rod-like shaped nanoparticle with a diameter of 18 nm and length of 300 nm. The TMV nanoparticle consists of a capsid with 2130 identical coat protein subunits, which are responsible for assembling into a helical structure around the ssRNA. When each subunit is modified, the resulting TMV is a polyvalent nanoparticle. The TMV can withstand temperatures up to 60 °C and can be stable in a pH range of 2–10. The TEM micrograph of wild type TMV is shown in Figure 2a [56].

In the human body, bone tissue regenerates to a greater extent when compared to other types of tissues. However, the regeneration process is complicated in the case of tumor resection, hip implant revision, and major fractures [63]. Pi et al. constructed a cartilage targeting gene delivery nanocomposite system by conjugating polyethylenimine (PEI) with M13 phage-displayed chondrocyte-affinity peptide (CAP), DWRVIIPPRPSA, which was isolated after two rounds of biopanning. During incubation, the phages expressing CAP showed higher affinity towards rabbit chondrocytes at 265.5-fold when compared to unmodified phages. They reported that the CAP-conjugated PEI particles had no species specificity in binding chondrocytes of rabbit and humans. Furthermore, most of the particles were found to enter chondrocytes without being trapped in ECM, which acknowledges their larger transfection efficiency [64].

T7 viral nanoparticles were explored to display two different functional peptides CARSKNKDC (CAR) and CRKDKC (CRK) to target the microvasculature of regenerating wound tissue, including skin and tendon [25]. Skin disintegration may occur in many ways, such as bruising, abrasion, hacking, burning, stabbing, and laceration. It was observed that CAR was similar to heparin-binding sites, whereas CRK was homologous to a segment of thrombospondin type I repeat. Interestingly, CAR displayed a dominant function in the early stages of skin wound healing, while CRK showed preferences in the later stages of the same process. As the terminal residues contain cysteine, the screened peptides had more feasibility to be involved in disulfide bond formation to form a molecular cycle structure. The CAR-expressing T7 phage nanoparticles had been found to appear in wound sites 100–140-fold more efficiently than the non-recombinant phage nanoparticle [65]. The biomedical application of siRNAs is minimal owing to their low absorption across the stratum corneum, a horny outer layer of skin. Hsu et al. [66] explored M13 phage (from Ph.D-C7C library) viral nanoparticle-expressing skin penetrating and cell entering (SPACE) peptide with the sequence of AC-KTGSHNQ-CG in order to reach therapeutic macromolecules, including siRNAs, into the skin-associated cells. The in vitro physicochemical studies explored that the various macromolecules, including siRNA, penetrated across the stratum corneum into the epidermis layer of skin through the macropinocytosis pathway when the molecules were conjugated with SPACE.

A muscle binding M13 phage nanoparticle with peptide sequence ASSLNIA was identified to possess more excellent selectivity (at least five-times more) compared to the control phage nanoparticle. While investigating overall muscle selectivity on different organs, the muscle binding affinity was found to be 9–20-fold for the skeletal and 5–9-fold for cardiac muscle [67]. Sun et al. synthesized functional multivalent M13 phage (Ph.D.-7^TM^ display library) nanoparticles to express RIYKGVIQA and SEEL sequences, which are found in Nogo-66, a neurite outgrowth inhibitory protein. They selectively bound negative growth regulatory protein 1 (NgR1) with electrostatic forces of repeated leucine residues, enhancing neural differentiation of pc12 cells. Hence, this specific engineered viral nanoparticle has been appreciated for its potential use in neurite tissue regeneration, including spinal cord injury, optic nerve injury, ischemic stroke, and neurodegenerative diseases [68]. Collett et al. suggested that hepatitis C virus-based nanoparticles could act as a quadrivalent vaccine to trigger humoral and cellular immune responses. They explored biophysical, biochemical, and biomechanical properties of nanoparticles using Atomic Force Microscopy and observed that glycosylation occurred on the surface of the nanoparticle with ordered packing of the core [69]. The literature reports revealed that Sendai virus vectors displaying cardiac transcription factors could efficiently reprogram both mouse and human fibroblasts into induced cardiomyocyte-like cells in vitro. In addition, they could reduce scar formation, maintaining cardiac function in myocardial infarction affected animals [70].

**Figure 3:**
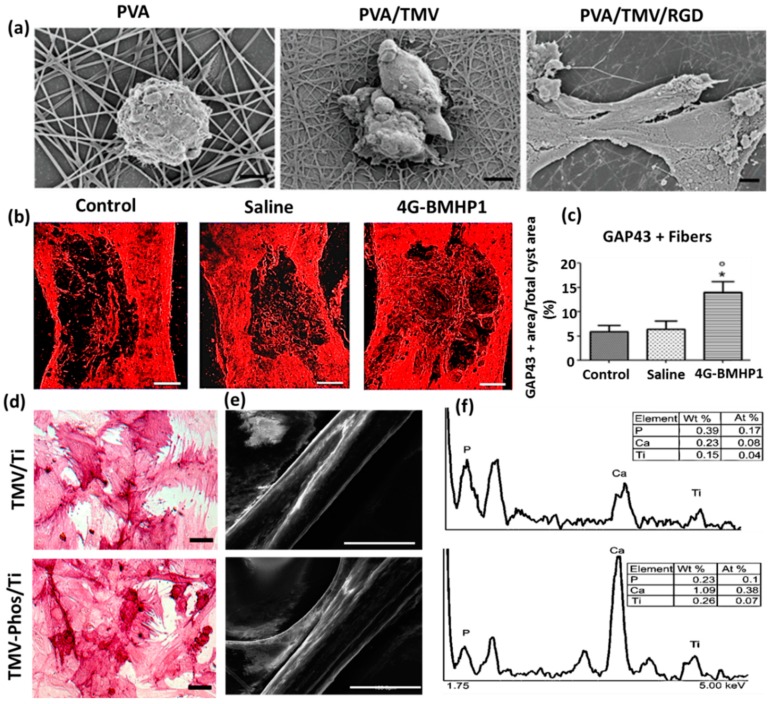
(**a**) Field emission scanning electron micrographs of Baby Hamster Kidney cells after 1 h incubation on electrospun nanofibrous substrates PVA, PVA-TMV, and PVA-TMV/RGD. Scale bar 2 μm. Reproduced with permission from [71]. Copyright John Wiley and Sons, 2014. (**b**) Immunofluorescence staining of longitudinal sections of total cyst area in spinal cord injury (SCI)-treated female Sprague-Dawley rats during the chronic phase. The synthesis of GAP-43 immunopositive fibers was significantly greater in bone marrow homing peptide-expressed phages (4G-BMHP1) treated group when compared to control groups (SCI control and saline). Scale bar 400 mm. The percentage of immunopositive fibers into the cyst area per total cyst area is represented in a bar graph (**c**) from six independent experiment results, and the values are reported with ± SEM. Significant factor * *p* ˂ 0.05, 4G-BMHP1 vs. SCI control; 4G-BMHP1 vs. saline. Reproduced with permission from [72]. Copyright PLOS, 2011. (**d**) Bright-field optical microscope images of histochemical staining of alkaline phosphatase (ALPL) in bone marrow-derived stem cells (BMSCs) in TMV and phosphate grafted TMV (TMV-Phos)-coated Ti substrates after 14 days of culture. BMSCs have been shown to form neighboring well-spread cells under osteogenic conditions, which are stained highly positive for ALPL. Scale bar is 100 μm. (**e**,**f**) SEM micrographs and EDX analyses of TMV and TMV-Phos coating on Ti substrates. Scale bar for SEM is 100 μm. Reproduced with permission from [73]. Copyright Elsevier, 2010.

The phosphate tailored TMV nanoparticle was demonstrated to induce expression of osteospecific genes of rat bone marrow stem cells (BMSCs), including osteocalcin and osteopontin, when compared to unmodified TMV nanoparticles. As shown in Figure 3d–f, the enhanced cell attachment and spreading of BMSCs were observed in phosphate grafted TMV (TMV-Phos) coated Ti substrates more than TMV coated substrates after 14 days of incubation in cell culture [73].

### 2.2. Virus-Incorporated 2D Films and Nanofibers

A combinatorial biomaterial consisting of PVX-based cyclic RGD, containing filament (RGD-PVX) and polyethylene glycol conjugated stealth filament (PEG-PVX), was developed to analyze biodistribution in mice xenograft models. The comparative studies demonstrated that PEG-PVX was preferentially accumulated into tumor cells, while RGD-PVX was trapped into the lung site in a large quantity. It has been reported that the filamentous and elongated nanoparticles are more advantageous in drug targeting than the spherical counterparts. Non-spherical nanoparticles present more ligands on their surfaces and show significant accumulation towards the vessel wall, improving the efficiency of tumor homing. Owing to the flexible nature of viral capsid, PVX-based nanoparticles could pass through restrictions in the complex biological environments and permeate into tissue cells without difficulty [74]. Wu et al. [56] successfully synthesized TMV-based electroactive nanofibers for neural tissue regeneration from the blend of polyaniline (PANI) and sodium polystyrene sulfonate (PSS). The morphology of the TMV/PANI/PSS nanofiber has been shown through TEM micrograph in Figure 2b.

An electrospun nanofiber of blends of polyvinyl alcohol (PVA) and TMV/RGD afforded higher cell density of baby hamster kidney (BHK) cells in culture. The enhanced cell adhesion and spreading and the formation of F-actin filaments were observed more on PVA/TMV/RGD nanofiber than PVA and PVA/TMV nanofibrous substrates, which were noticed in SEM micrographs (Figure 3a). The resulting nanofiber provided electroactivity and topographical cues to the neural cells and was reported to augment the length of neurites, increase the population of cells, and lead the cellular bipolar morphology more than TMV-based non-conductive nanofibers [71]. Korehei et al. [57] produced a virus incorporated nanofiber by electrospinning the blends of polyethylene oxide (PEO) and T4 bacteriophage suspension. The SEM measurement showed that T4 bacteriophages were protected from severe electrospinning conditions, as they were concentrated within the alginate capsule, as can be seen in Figure 2c. The alginate beads containing phages were found to exhibit smooth rounded surfaces. The size of the electrospun nanofiber of PEO/alginate/T4 bacteriophages had an average diameter 500 ± 100 nm (Figure 2d). According to TEM measurement, the capsules of T4 bacteriophages were distributed without uniformity throughout the fiber matrix (Figure 2e).

The induced pluripotent stem cells (iPSCs) are a promising cell source, which can rise to different cell lineages and construct a well-developed functional bone substitute. However, there is a challenge in osteoblastic differentiation of iPSCs by a conventional biomaterial, as it may form teratoma, raising health risks. Wang et al. [75] demonstrated that a phage (M13)-based nanofiber with four different signal peptides aiming to influence stem cell fate could be potentially utilized for bone tissue regeneration. The aligned nanofibrous matrix provided biochemical and biophysical cues to the cells promoting differentiation of iPSCs into osteoblasts. Among the signal peptides investigated by them were two adhesive-directing peptides RGD and RGD/PHSRN from fibronectin, and the remaining two included ALKRQGRTLYGFGG and KIPKASSVPTELSAISTLYL sequences, which are the growth regulating peptides from osteogenic growth factor and bone morphogenetic protein 2 (BMP2), respectively. The layer-by-layer technique produced a phage-assembled nanofiber assuming nanotopography of the ridge-groove structure, wherein the phage strands were parallel to each other but separated by grooves. Due to this specialized nanotopography of material, the occurrence of controlled osteoblastic differentiation was observed, even in the absence of osteogenic supplements. The research group reported that the phages displaying growth factor signal peptides could express a higher level of alkaline phosphatase (ALP) than the phages having adhesive signal peptides on the surface. The in vivo animal studies disclosed that iPSCs alone caused teratoma after one month of cells injection into nude mice, whereas the group of iPSC-derived osteoblasts did not. Cigognini and co-workers engineered an electrospun nanofibrous scaffold dispersing phage-displayed bone marrow homing peptide (BMHP (1) with sequence PFSSTKT and investigated its potential use in a chronically damaged spinal cord, which was caused by the degeneration of the central nervous system [72]. The clinical data showed that the biomimetic material enhanced nervous tissue regeneration, owing to porosity and nanostructure at the microscopic level, and improved the locomotor recovery of experimental rats. From Figure 3b,c, the histological analyses revealed that the scaffold affected increased cellular infiltration and axonal regeneration after eight weeks of experimental investigation in rats. They found a higher synthesis of growth-associated protein 43 (GAP-43) in engineered scaffold-treated animal when compared to saline and control groups with spinal cord defects. Our research group has explored electrospun nanofibrous matrices of PLGA containing self-assembled M13 bacteriophages along with additives RGD and graphene oxide to show enhanced differentiation of fibroblasts, smooth muscle cells, and myoblasts [76,77,78,79,80].

### 2.3. Virus-Incorporated 3D Hydrogel Scaffolds

Cell-laden-agarose hydrogel was prepared by dispersing genetically engineered rod-shaped PVX nanoparticles, which present functional RGD peptides and mineralization inducing peptides (MIP) on its surface, into agarose polymeric components [81]. Luckanagul et al. [59] prepared freeze-dried solid foam of a porous alginate hydrogel (PAH) comprising TMV. The incorporation of TMV nanoparticles resulted in large sized and well-defined spherical pores (100–500 μm) in TMV/PAH, analyzed by Field Emission Scanning Electron Microscope image (Figure 2g).

The PVX nanoparticles adopted a nano-filamentous structural network on coated surfaces. Exploiting the synergistic effect of both peptides, the PVX nanoparticles in hydrogel expressed significant cell adhesion as well as hydroxyapatite nucleation. Confirmed by SEM and immunostaining characterizations, it was further reported that the viral nanoparticles could be preserved over 14 days in hydrogel and the whole biomaterial could act as a promising bone substitute. Maturavongsadit et al. [82] developed an injectable TMV based hydrogel under physiological conditions to imitate a cartilage microenvironment. The hydrogel was prepared by cross-linking methacrylate hyaluronic acid polymers by cysteine inserted TMV mutants involving in situ Michael addition reaction. The hydrogel was reported to influence enhancement of cartilage tissue regeneration by promoting chondrogenesis via up-regulation of BMP-2. The interaction of TMV nanoparticles with the cells assisted the high-level expression of BMP-2, an effective inducer of differentiation of mesenchymal stem cells into chondrocytes.

Luckanagul et al. [58] investigated the performance of functional TMV-RGD-blended alginate hydrogel nanocomposites to treat in vivo cranial bone defects in Sprague-Dawley rats. The TMV-functionalized sponge-like hydrogel supported cell localization without triggering any systemic toxicity in the defect area, and hence was envisaged as an active bone replacement biomimetic material in the future direction of reconstructive orthopedic surgery. Shah et al. [83] studied an integrated co-assembled hydrogel system of peptide amphiphiles, in which M13 phage coat protein was modified to express a high density of binding peptide HSNGLPL to combine with transforming growth factor β1 (TGF-β1). The research group found an enhancement in articular cartilage tissue regeneration in a rabbit model with a full-thickness chondral defect because of the slower release of growth factor from the hydrogel, with approximately 60% of cumulative drug release at 72 h, which supported the viability and chondrogenic differentiation of mesenchymal stem cells in the defective site. The in vivo evaluation of the rabbit model showed that the hydrogel treated animal group had no apparent symptoms of chronic inflammatory responses after four weeks. All of the rabbits appeared with a full range of motion in their knees at the end of the investigation. Caprini et al. [84] isolated M13 phage-displayed peptide, KLPGWSG, which could adhere on the surface of murine neural stem cells. Subsequently, the research group designed a self-assembled KLPGWSG-based biomimetic hydrogel with tunable visco-elastic properties for the regeneration of the degenerated nervous system. It was discovered that the phage-based hydrogel favored cell adherence and differentiation in the range of 100–1000 Pa, suggesting that the elastic property of the matrix is a crucial factor in tissue regeneration.

### 2.4. Virus-Incorporated Organic-Inorganic Hybrid Nanocomposites

The interaction of organic and inorganic biocompatible materials in scaffolds bring about significant impacts in biomedical applications. Cementum, classified as a hard mineralized tissue, surrounds tooth root and has been a part of periodontal tissue that connects the tooth to the bone. When an infectious biofilm adheres to tooth root, triggering periodontal disease, the tooth loss is more enhanced. Gungormus et al. [85] demonstrated amelogenin-derived M13 phage-displayed peptide controlled hydroxyapatite biomineralization for dental tissue regeneration. It was reported that Amelogenin directed hydroxyapatite to form a protein matrix during the formation of enamel. Hence, the research group synthesized the cementomimetic material by applying an aqueous solution of the amelogenin-displayed peptide on the human demineralized root surface to form a layer, which was subsequently immersed into the solution of calcium and phosphate ions. Ramaraju et al. [86] isolated M13 phage-displayed peptides to design a dual functional apatite-coated film for effective bone tissue regeneration. They reported that one peptide sequence of the phage, VTKHLNQISQSY, had mineral (apatite) binding affinity with 25% hydrophobicity, whereas another peptide, DPIYALSWSGMA, had cell binding affinity with 50% hydrophobicity. Also, they discovered that the dual functional apatite-based biomaterial could stimulate the adhesion strength of human bone marrow stromal cells (hMSC) and subsequently increase cell proliferation and differentiation. Due to the mineral binding affinity, the film provided a platform for the adherence of osteogenic cells with osteoconductive and osteoinductive signals. Further, the biomimetic nanocomposite showed a greater extent of proliferation of hMSCs with an elevated level of Runx2 expression when compared to biomimetic apatite without functional peptides.

Wang et al. [58] prepared a 3D-printed biomimetic nanofiber with M13 phage-displayed RGD peptides residing in the pores of the scaffold to enhance bone tissue regeneration. The nanocomposite consisted of hydroxyapatite and tri-calcium phosphate showing an ordered pattern with interconnected micro and macro scale pores, which are shown in the TEM micrograph (Figure 2f). The research group implanted a MSC-seeded biomimetic scaffold into a rat radial bone defect and discovered that the order of regeneration was found as follows: scaffold filled with modified phages > scaffolds filled with wild-type phages > pure scaffold. He et al. [60] carried out a similar kind of research work, genetically modifying M13 phage to express oligonucleotide encoding E8 and inducing self-assembly followed by oriental mineralization to synthesize nanofibrous biomimetic materials under the influence of divalent calcium ions. The resulting mineralized phage bundle has been shown in TEM micrography (Figure 2h). Wang et al. [61] used Ca^2+^ ions to prompt self-assembly of fd phage-based anionic nanofibers and transform them into a bundle sheet (Figure 2i), which provided insights into biomineralization and fabrication of organic–inorganic hybrid nanocomposites. The divalent ion-triggered bundle not only acted as a biotemplate but also served as a Ca source to initiate the ordered nucleation and growth of crystalline hydroxyapatite in the biological fluid.

## 3. Other Formulations of Virus-Based Nanocomposites with Different Biomedical Applications

Apart from tissue regeneration, virus-based biomimetic nanocomposites have traced their steps in different biomedical applications, such as drug delivery, bioimaging, and biosensing. Wang et al. [87] studied f8/8 phage-based polymeric micelles from the self-assembly of polymeric PEG- diacyl lipid conjugates. These polymeric micelles were reported to have cell-targeting ability to release less water-soluble drugs with more specificity towards breast cancer Michigan Cancer Foundation-7 (MCF-7) cells. The non-toxic filamentous f88.4 bacteriophage viral nanoparticle, which was designed to display a single chain antibody, delivered the vectors to the different regions of the brain in albino, laboratory-bred nude mice (BALB/c), and hence was proposed for treating Alzheimer’s disease with early diagnosis [88,89]. Wang et al. [90] studied a M13 phage-displayed peptide with the sequence HSQAAVP to target fibroblast growth factor 8b (FGF8b) to treat prostate cancer. The genetic level disturbances in homeostasis between prostate epithelial and stromal cells cause prostate cancer. The major isotherm of fibroblast growth factor 8 is FGF8b, which is associated with the stages of prostate cancer and has been a potential target for appropriate therapies. In this study, the research group revealed that the biomimetic material interrupted FGF8b binding to its receptors, and thereby prevented FGF8b-induced cell proliferation. Furthermore, they reported that the biomaterial had the potential to arrest the cell cycle at the phase G0/G1 by suppressing cyclin D1 and proliferating cell nuclear antigens (PCNA).

Carrico et al. [43] chemically modified the amino acid residues present on the surface of filamentous fd phage coat protein following a two-step transamination/oxime reaction for its potential use in characterizing breast cancer cells. The research group discovered that the chemical reaction selectively targets N-terminal groups but is not involved in transamination of lysine ε-amines. They conjugated PEG polymeric chains to the phage protein in order to reduce immunogenicity, decrease non-specific binding, and increase solubility in the aqueous environment. They observed that there were no significant differences in either absorption or emission properties after fluorophores were labeled with polymer conjugated phages. Fan et al. [91] isolated cyclic peptide CAGALCY from T7 phage nanoparticles in order to target the pial microvasculature of the brain and inhibit platelet adhesion. The presence of the bulky hydrophobic core, two cysteine residues at each end, and the tyrosine residue at the carboxy terminus are considered as remarkable features for selectively binding the brain microvasculature. When pharmacokinetic properties were assessed, the non-filamentous phage, T7, showed a fast clearance rate from the blood with a half-life of 12 min, whereas the filamentous phages M13 and fUSE5 had longer half-lives of 7 h and 9 h, respectively. To identify the specificity of the T7 phage-displayed peptide, they determined selectivity indices using plaque assay for various organs of mice, including lung, liver, brain, kidney, colon, small intestine, and large intestine. The characterization results exposed that T7 displayed peptide resided (accumulated) in the brain, with a selectivity index of 1000, whereas other organs possessed low specificity for the peptide, with selectivity indices less than 50.

Bean et al. [92] prepared a bacteriophage K (ΦK) by incorporating the virus into a photo cross-linked hyaluronic acid methacrylate (HAMA)-based hydrogel that resulted in a material with antimicrobial properties. The presence of two zinc finger genomes (CX_2_CX_22_CX_2_C and CX_2_CX_23_CX_2_C) in the virus caused it to be virulent against a wide range of infective Staphylococci. The secretion of hyaluronidase enzyme-mediated *S. aureus* sensitizes HAMA and triggered degradation of the hydrogel, facilitating the release of ΦK at a sustained level to inhibit bacterial growth effectively. This stimuli-responsive hydrogel was shown to reduce pain, promote cell migration and tissue hydration in the wound site, and was suggested for the application of dermal tissue regeneration. Schmidt et al. [93] identified two different adenovirus phage-displayed peptides QTRFLLH and VPTQSSG to target neural precursor cells (NPC) in the hippocampal dentate gyrus of adult mice through adenovirus-mediated gene transfer. The peptides were found to be strongly internalized into NPCs when the investigated material was added to neurosphere culture containing clusters of neural stem cells.

Kelly KA et al. [94] isolated high-throughput fluorochrome-labeled M13 phage particles (Ph.D. C7C library) to rapidly identify ligands of biological interest in vivo using secreted protein acidic and rich in cysteine (SPARC) molecules and vascular cell adhesion molecules-1 (VCAM-1). The engineered phage particles led to higher sensitivity with an attachment of 800 fluorophores per phage. Wan et al. [37] developed an f8/8 phage-based biosensor exploiting magnetoelastic wireless detection system. The genetically modified phage-expressed peptide sequence EPRLSPHS on the surface of the target biological agent, *Bacillus anthracis* spore. The resonance frequency of the sensor decreased gradually depending on the binding agent on the surface. They reported that this affinity-based phage-displayed biosensor exhibited more longevity activity as a diagnostic probe to target numerous agents with more efficiency than antibody-based biosensors.

## 4. Conclusions and Perspectives

The potential application of virus-incorporated biomimetic nanocomposites in the form of self-assembled nanoparticles, nanofibers, hydrogels, and organic–inorganic hybrids in the field of tissue regeneration has been elucidated in this review. Though virus-based biomaterial has displayed many beneficial properties, there are some issues to be addressed. (1) Many research groups have expressed desired peptides on the surface of phage-based viral nanoparticles exploiting phage libraries. However, whether the number of peptides exhibited by each nanoparticle is the same is questionable. (2) Biodistribution of viral nanoparticles in different organs of animal tissues has been studied by some researchers. Still, a comprehensive study to describe bioavailability must be demonstrated. (3) It has been well documented that viral nanoparticles contribute to the enhancement in tissue regeneration. However, a systematic study is required to explain the phases of tissue regeneration, in which viral nanocomposites contribute more. (4) The viral nanocomposites in the form of polymeric micelles, vesicles, and dendrimers are less formulated and have not been explored enough for the application of tissue regeneration. The following are suggestions for the future of this field. (1) Sophisticated techniques and methodology to quantify the number of peptides expressed on each phage particle. (2) Pharmacokinetic and pharmacodynamics studies to determine the required dosage of viral nanoparticles in each organ type of tissue regeneration. (3) An extensive in vivo animal study to show the influence of viral-based nanocomposites in each phase of tissue regeneration. (4) Successful bioconjugation of viral nanoparticles with amphiphilic polymers or surfactants to design various oil-in-water-type emulsions. We hope that the researchers with interdisciplinary backgrounds will advance the field of tissue regeneration using viral-based biomimetic nanocomposites by considering the problems and the concerned suggestions.
